# Clinical significance of potential drug–drug interactions in a pediatric intensive care unit: A single-center retrospective study

**DOI:** 10.1371/journal.pone.0246754

**Published:** 2021-02-08

**Authors:** Yu Hyeon Choi, In Hwa Lee, Mihee Yang, Yoon Sook Cho, Yun Hee Jo, Hye Jung Bae, You Sun Kim, June Dong Park

**Affiliations:** 1 Department of Pediatrics, Seoul National University Hospital, Seoul National University College of Medicine, Seoul, Korea; 2 Department of Pharmacy, Seoul National University Hospital, Seoul, Korea; Kaohsuing Medical University Hospital, TAIWAN

## Abstract

Despite the high prevalence of potential drug–drug interactions in pediatric intensive care units, their clinical relevance and significance are unclear. We assessed the characteristics and risk factors of clinically relevant potential drug–drug interactions to facilitate their efficient monitoring in pediatric intensive care units. This retrospective cohort study reviewed the medical records of 159 patients aged <19 years who were hospitalized in the pediatric intensive care unit at Seoul National University Hospital (Seoul, Korea) for ≥3 days between August 2019 and February 2020. Potential drug–drug interactions were screened using the Micromedex Drug-Reax^®^ system. Clinical relevance of each potential drug–drug interaction was reported with official terminology, magnitude of severity, and causality, and the association with the patient’s clinical characteristics was assessed. In total, 115 patients (72.3%) were exposed to 592 potential interactions of 258 drug pairs. In 16 patients (10.1%), 22 clinically relevant potential drug–drug interactions were identified for 19 drug pairs. Approximately 70% of the clinically relevant potential drug–drug interactions had a severity grade of ≥3. Exposure to potential drug–drug interactions was significantly associated with an increase in the number of administrated medications (6–7 medications, *p =* 0.006; ≥8, *p<*0.001) and prolonged hospital stays (1–2 weeks, *p =* 0.035; ≥2, *p =* 0.049). Moreover, clinically relevant potential drug–drug interactions were significantly associated with ≥8 prescribed drugs (*p =* 0.019), hospitalization for ≥2 weeks (*p =* 0.048), and ≥4 complex chronic conditions (*p =* 0.015). Most potential drug–drug interactions do not cause clinically relevant adverse outcomes in pediatric intensive care units. However, because the reactions that patients experience from clinically relevant potential drug–drug interactions are often very severe, there is a medical need to implement an appropriate monitoring system for potential drug–drug interactions according to the pediatric intensive care unit characteristics.

## Introduction

Coadministration of two or more drugs is associated with a potential drug–drug interaction (PDDI), which is the possibility that the drugs alter each other’s effect [1]. Critically ill patients are at a higher risk of drug-drug interactions (DDIs), not only due to multiple medications but also because of disease complexity, accompanying organ dysfunction, and pharmacotherapy complexity [2]. Moreover, adverse drug reactions (ADRs) including DDIs in ICU are highly associated with prolonged hospitalization and higher morbidity and mortality [3]. With increasing concerns regarding medication safety in intensive care units (ICUs), numerous studies have reported PDDIs over the past decades. According to a recent meta-analysis, the proportion of adult patients in ICUs with exposure to at least one PDDI is 58%, which is higher than that in general wards [4]. In pediatric intensive care units (PICUs), the overall prevalence of PDDIs is 59.4%–75.2%, similar to that in adult ICUs [5,6].

PDDIs do not always result in adverse events or actual harm because some drugs are coadministered intentionally with favorable effects in ICUs. For example, opioids and benzodiazepine are concomitantly administered for analgosedation as a component of basic pain management in ICUs [7]. In addition, some medications must be coadministered despite well-known interactions. Therefore, the assessment of DDI-related negative effects or ADR intensity can help clinicians identify drug combinations that should be avoided [8]. Nevertheless, there are only a few studies that assessed the clinical relevance of PDDIs and PDDI-related ADRs without mentioning causality and severity [6,9].

In this study, we aimed to assess clinically relevant (CR)-PDDIs in PICUs, excluding common PDDIs without clinical significance. Specifically, we focused on providing guidance for efficient PDDI monitoring in PICUs by identifying the characteristics and risk factors of CR-PDDIs in this critical care setting.

## Methods

### Data sources and eligibility criteria

This retrospective study was conducted in the PICU at the Seoul National University Hospital (Seoul, Republic of Korea), which is a mixed unit for medical and surgical pediatric patients. The unit has 24 beds and is staffed 24 h a day, 7 days a week, by four pediatric intensivists. Clinical pharmacy services are provided 5 days a week by a clinical pharmacist, who reviews the medication records, including drug dosing, administration route, drug concentration, reported ADRs, and any drug-related queries, of all PICU patients [10]. A clinical pharmacist was assigned to stay in the PICU for 4 h each day for dedicated jobs. PDDIs in PICU patients were monitored from March 2019. The assigned pharmacists recorded clinical interventions and the associated outcomes in a pharmaceutical care database.

**Fig 1:**
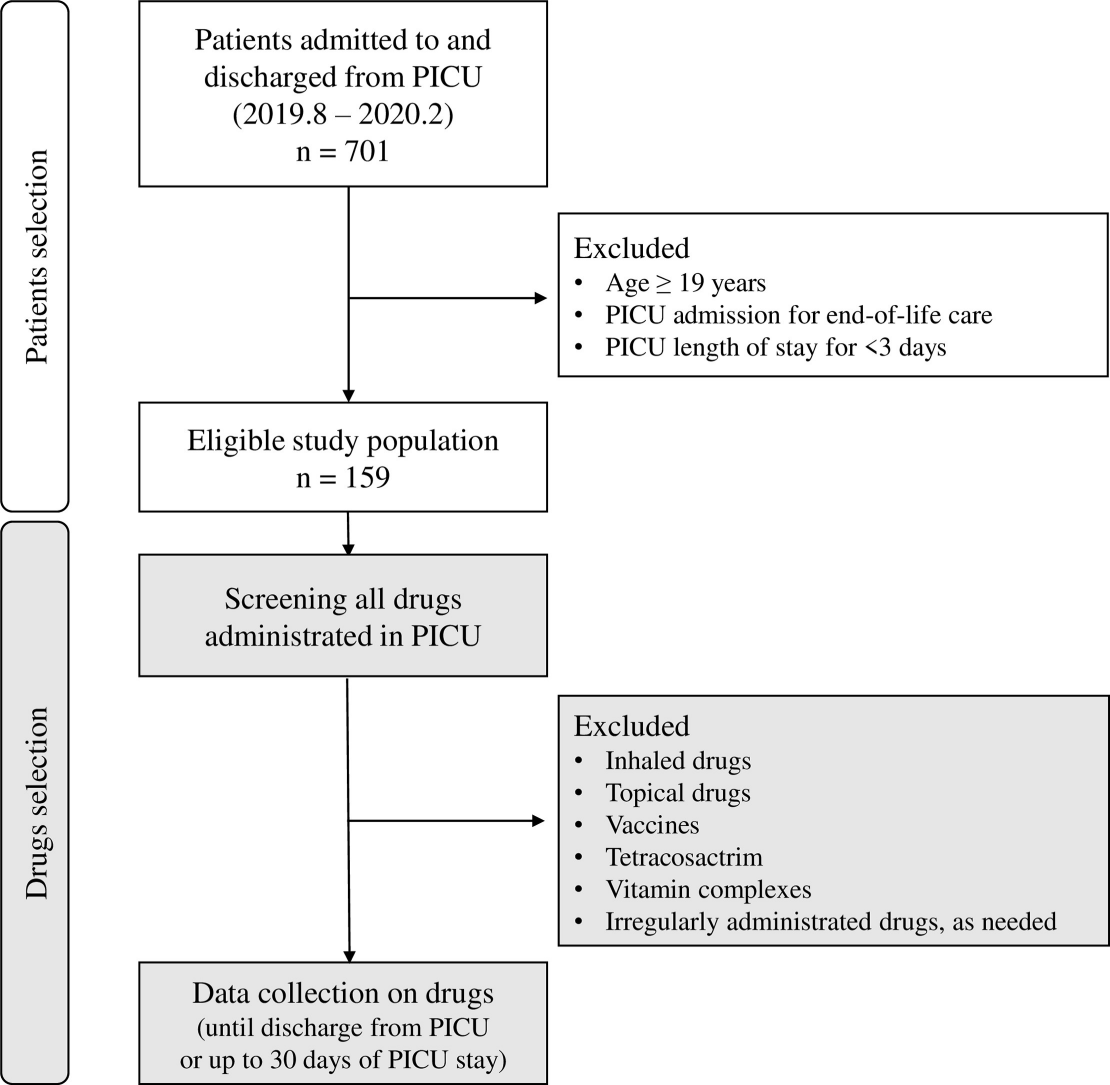
Flow chart of the study protocol.

The data of all patients aged <19 years who were admitted to and discharged from the PICU between August 2019 and February 2020 were analyzed. We excluded patients who were admitted for end-of-life care and those who stayed in the PICU for <3 days (Fig 1). This minimum length of PICU stay was adopted to ensure sufficient observation time for identifying CR-PDDIs. The study was approved by the Institutional Review Board (IRB No. C-1908-058-1054) at the Seoul National University Hospital. The requirement to obtain informed consent from patients was waived.

Data were retrospectively compiled from electronic medical records into a structured data collection form as follows: patients’ demographics (including age, sex, and major diagnosis), department at admission, cause of PICU admission, predicted mortality rate using the prediction algorithm of Pediatric Risk of Mortality IV to assess severity at admission, length of PICU stay, and PICU outcome. Additionally, the accompanying complex chronic conditions (CCCs) that each patient presented during PICU admission were identified according to the updated pediatric CCC classification system, version 2 [11]. We established the age groups according to the criteria suggested by the Ministry of Food and Drug Safety at the International Conference on Harmonisation, and the definition of pediatric sepsis [12,13].

We obtained data regarding all drugs administered to the eligible patients until discharge from PICU or 30 days after PICU admission, whichever occurred first (Fig 1). The drug data included generic name, precise administration timing, and administration route. The following drugs were excluded because they are associated with less systemic drug interactions than other drugs: 1) inhaled drugs, such as salbutamol, ipratropium, budesonide, acetylcysteine, and epinephrine; 2) topical drugs; 3) vaccines; 4) tetracosactrin for adrenal function test; 5) vitamin complexes; and 6) any drug administered irregularly and intermittently as needed, such as diuretics, antipyretics, and analgesics (drugs that were not administered according to a regular dosing interval or on consecutive days). Administration of drugs within a 24-h period were regarded as concurrent exposure. However, if one drug was changed to another, it was not regarded as coadministration.

### Identification of PDDIs

PDDIs were reviewed using the Micromedex Drug-Reax^®^ system (Truven Health Analytics, Greenwood Village, CO, USA). Several software packages are available for screening PDDIs, but this software has been validated and found to be superior to others. In the present study, we used it for screening PDDIs in the PICU [5,6,14,15]. During the study period, there were 14 drugs that could not be searched in the Micromedex Drug-Reax^®^ system; therefore, these drugs were excluded (S1 Appendix). The PDDI classifications were based on severity (contraindicated, major, moderate, and minor) and documentation (excellent, good, and fair). A “contraindicated” drug combination should never be used concurrently. “Major” interactions could be life-threatening and/or require medical intervention to minimize or prevent severe adverse effects. “Moderate” interactions could result in exacerbation of the patient’s condition and/or require treatment alteration. “Minor” interactions had limited clinical effects that could be included in the frequency or severity of adverse effects, but generally, they do not require a major treatment alteration. At the documentation level, “excellent” indicated that controlled studies had clearly established the existence of a drug interaction. “Good” indicated that documentation strongly suggested an interaction, but well-controlled studies were lacking. “Fair” indicated that available documentation was poor, although pharmacologic considerations led clinicians to suspect an interaction.

### Assessment of CR-PDDIs

The clinical relevance of each PDDI was thoroughly reviewed by two authors (YHC and JDP), based on the changes in vital signs, laboratory test results, electrocardiogram, and judgment of the assigned doctor. To clarify clinical relevance, only DDIs causing ADRs that altered the treatment course and interventions of any form were regarded as CR-PDDIs. The development of CR-PDDIs was observed until discharge from the PICU or 30 days after PICU admission, whichever occurred first.

CR-PDDIs were reported according to the causality and magnitude of ADRs resulting from DDIs. The causality of events was classified into certain, probable/likely, possible, unlikely, conditional/unclassified, and unassessable/unclassifiable, according to the system proposed by the World Health Organization Collaborating Center for International Drug Monitoring, the Uppsala Monitoring Center [16]. DDIs causing ADRs with a high causality, such as certain, probable/likely, and possible, were regarded as CR-PDDIs. The terminology and severity of CR-PDDIs were evaluated based on the Common Terminology Criteria for Adverse Events (CTCAE) v5.0 [17].

### Statistical analysis

Continuous nonparametric data are presented as median with interquartile range and categorical variables as number with percentage. Logistic regression was performed to identify variables that were significantly associated with any PDDI and CR-PDDI. Patient characteristics such as sex, age, department, CCC number, PICU admission reasons, predicted mortality rate at admission, average prescribed medication number, and PICU stay duration were independent variables in the model. All variables were included in analyzing the predictors of CR-PDDIs and PDDIs. However, when identifying associations of three variables (CCC number, prescribed medication number, and length of PICU stay) with a CR-PDDI, the top two of three divided groups were merged because of small populations in each subgroup. For each variable, the odds ratio (OR) and 95% confidence interval (CI) were determined. Variables with a significant univariate result (*p<*0.05) were included in the multivariate logistic regression analysis. Results with *p≤*0.05 were considered statistically significant. Statistical analyses were performed using SPSS software (version 25; SPSS Inc., IBM, Armonk, NY, USA).

## Results

### General characteristics

Data of 159 pediatric patients admitted to the PICU were analyzed (Table 1). The median age of the children was 0.92 (0.33–5.25) years, and 96 patients (60.4%) were aged <2 years. Approximately 40% of the patients belonged to the general pediatric department, and the median CCC number was 2 (1–3). The most common reason for PICU admission was postoperative/procedure care (90, 56.6%), followed by respiratory failure (33, 20.8%). The patients in the PICU received an average of 8.0 (5.3–10.5) medications and were exposed to an average of 1.2 (0.0–2.5) PDDIs. The median length of PICU stay was 6.1 (4.0–11.5) days per patient.

**Table 1:** General characteristics of the patients included (n = 159).

Characteristic	n (%) or median (IQR)
Sex	
Male	91 (57.2)
Female	68 (42.8)
Age (years)	0.9 (0.3–5.3)
0–27 days	23 (14.5)
28 days–23 months	73 (45.9)
2–5 years	27 (17.0)
6–11 years	14 (8.8)
12–18 years	22 (13.8)
Department	
General pediatrics	63 (39.6)
Pediatric cardiology	11 (6.9)
Thoracic surgery	45 (28.3)
General and neurosurgery	40 (25.2)
Diagnosis	
Cardiovascular disease	55 (34.6)
Haemato-oncology disease	24 (15.1)
Neurologic disease	23 (14.5)
Gastrointestinal disease	23 (14.5)
Respiratory disease	15 (9.4)
Other diseases^a^	19 (11.9)
Number of complex chronic conditions	2 (1–3)
Reason for PICU admission	
Post-operative/procedure care	90 (56.6)
Respiratory failure	33 (20.8)
Shock	10 (6.3)
Mental change	7 (4.4)
For continuous renal replacement therapy	6 (3.8)
Other causes	13 (8.2)
Predicted mortality rate at admission (%)	1.9 (0.9–7.3)
Average number of prescribed medications per patient^b^	8.0 (5.3–10.5)
Average number of PDDIs exposed per patient^b^	1.2 (0–2.5)
Length of stay in PICU (days)	6.1 (4.0–11.5)
Survival to PICU discharge	150 (94.3)

IQR, interquartile range; PICU, pediatric intensive care unit; PDDI, potential drug–drug interaction.

^a^Included other congenital/genetic defects, metabolic diseases, and renal/urologic diseases.

^b^Observed during admission in pediatric intensive care unit (maximum 30 days).

### PDDI exposure

In total, 115 patients (72.3%) were exposed to 592 PDDIs of 258 drug pairs. According to severity, 2.6%, 56.2%, and 39.0% of the 592 PDDIs were classified as contraindicated, major, and moderate, respectively, but based on documentation, 7.0%, 41.9%, and 52.3% were classified as having excellent, good, and fair scientific evidence, respectively. The frequency of the 258 drug pairs that caused PDDIs according to severity was as follows: contraindicated (6, 2.3%), major (141, 54.7%), and moderate (98, 38.0%). All patients exposed to PDDIs experienced one or more PDDI(s) with moderate or greater severity.

The most frequently identified PDDIs classified based on severity are shown in Table 2 (only those with approximately 1% prevalence). Midazolam + remifentanil, enalapril + spironolactone, and enalapril + furosemide were the most common combinations causing PDDIs. Furthermore, most of the common drug pairs resulting in PDDIs were thoroughly monitored by routine or intensive care in the PICU.

**Table 2:** Most frequently identified potential drug–drug interactions based on severity and their associated monitoring methods in the pediatric intensive care unit.

Severity	Drug pair	Evidence	Frequency	Potential adverse events	Monitoring methods
Contraindicated	Nitroglycerin + sildenafil	Excellent	5	Potentiation of hypotensive effects	Blood pressure
Major	Midazolam + remifentanil	Fair	40	Increased risk of hypoventilation	Respiratory rate, SpO_2_
	Enalapril + spironolactone	Good	33	Hyperkalemia	Electrolyte
	Piperacillin/tazobactam + vecuronium	Good	17	Enhanced and/or prolonged neuromuscular blockade	N/A
	Piperacillin/tazobactam + vancomycin	Good	10	Increased risk of acute kidney injury	Vancomycin trough level
	Midazolam + phenobarbital	Fair	8	Increased risk of hypoventilation	Respiratory rate, SpO_2_
	Famotidine + tacrolimus	Fair	7	Increased tacrolimus toxicity	Tacrolimus level, ECG
	Potassium chloride + spironolactone	Fair	7	Hyperkalemia	Electrolyte
	Aspirin + furosemide	Good	7	Reduced diuretic effectiveness, nephrotoxicity	Urine output, Cr
	Aspirin + spironolactone	Good	7	Reduced diuretic effectiveness, hyperkalemia, nephrotoxicity	Urine output, Electrolyte, Cr
	Vecuronium + nicardipine	Good	6	Enhanced neuromuscular blockade	N/A
	Phenobarbital + remifentanil	Fair	6	Increased risk of CNS depression	Assessment of sedation level^a^
	Famotidine + fluconazole	Fair	5	Increased fluconazole toxicity	ECG
Moderate	Enalapril + furosemide	Good	33	Postural hypotension (first dose)	Blood pressure
	Furosemide + vecuronium	Good	13	Increased or decreased neuromuscular blockade	N/A
	Furosemide + sildenafil	Fair	12	Increased risk of hearing loss	N/A
	Esomeprazole + levothyroxine	Good	6	Decreased levothyroxine effectiveness	Thyroid hormone test
	Aspirin + enalapril	Excellent	6	Decreased effectiveness of enalapril	Blood pressure
	Esomeprazole + fluconazole	Fair	5	Increased esomeprazole plasma concentrations	N/A
	Esomeprazole + iron	Fair	5	Reduced iron bioavailability	Hemoglobin, iron level
	Lansoprazole + iron	Fair	5	Reduced iron bioavailability	Hemoglobin, iron level
	Bosentan + sildenafil	Excellent	5	Increased bosentan plasma concentrations and decreased sildenafil plasma concentrations	Liver function test, Echocardiography

CNS, central nervous system; ECG, electrocardiogram; SpO_2_, percutaneous arterial oxygen saturation; Cr, creatinine; N/A, not available.

^a^Sedation level was assessed by State Behavioral Scale based on protocol in our PICU.

The PDDI-causing drug combinations frequently documented by the different departments are shown in Table 3. In pediatric cardiology and thoracic surgery, interactions of enalapril + spironolactone and enalapril + furosemide were observed in more than half of the patients (58.9%). In general pediatrics, midazolam + remifentanil was the most common PDDI-causing combination used in approximately 30% of the patients, whereas each of the other drug combinations associated with PDDIs was coadministered to a small proportion of patients.

**Table 3:** Most frequently identified potential drug–drug interactions by department.

Drug–drug combination	Number of patients exposed (%)
**General pediatrics (n = 63)**	
Midazolam + remifentanil	19 (30.2)
Piperacillin/tazobactam + vecuronium	6 (9.5)
Furosemide + sildenafil	6 (9.5)
Midazolam + phenobarbital	6 (9.5)
Furosemide + vecuronium	5 (7.9)
Piperacillin/tazobactam + vancomycin	5 (7.9)
Potassium chloride + spironolactone	5 (7.9)
Vecuronium + nicardipine	5 (7.9)
Bosentan + sildenafil	5 (7.9)
**Pediatric cardiology and thoracic surgery (n = 56)**	
Enalapril + spironolactone	33 (58.9)
Enalapril + furosemide	33 (58.9)
Midazolam + remifentanil	15 (26.8)
Aspirin + furosemide	7 (12.5)
Aspirin + spironolactone	7 (12.5)
Piperacillin/tazobactam + vecuronium	6 (10.7)
Furosemide + sildenafil	6 (10.7)
Aspirin + enalapril	6 (10.7)
Furosemide + vecuronium	5 (8.9)
**General and neurosurgery (n = 40)**	
Midazolam + remifentanil	6 (15.0)
Piperacillin/tazobactam + vecuronium	5 (12.5)
Famotidine + tacrolimus	4 (10.0)
Esomeprazole + levothyroxine	4 (10.0)
Furosemide + vecuronium	3 (7.5)

### Causality and severity of CR-PDDIs

Twenty-two clinically relevant adverse events related to PDDIs were identified in 16 patients (10.1%). There were 19 drug pairs associated with CR-PDDIs (Table 4), and 5 of those were combinations related to frequent PDDIs. The causality was classified as probable for 54.5% of the total CR-PDDIs and as possible for the remaining CR-PDDIs. There was no case of mortality, but a severity of grade ≥3 was confirmed for 15 (68.1%) CR-PDDIs. Two drug pairs associated with CR-PDDIs could not be assessed for causality and severity according to the CTCAE because of a lack of data.

**Table 4:** Clinically relevant potential drug–drug interactions: adverse event, causality, severity, and management.

Drug pair	Observed adverse events	Causality	Severity grade (frequency)	Incidence (%)^c^	Management
Enalapril + spironolactone^a^	Hyperkalemia	Probable	Grade 2 (1) Grade 3 (2)	9.1	Spironolactone discontinuation
Levofloxacin + pentamidine	Electrocardiogram QT corrected interval prolonged	Probable	Grade 4 (1)	100	Levofloxacin discontinuation
Defibrotide + nadroparin	Bronchopulmonary hemorrhage	Probable	Grade 3 (1)	25	Nadroparin discontinuation, transfusion
Esomeprazole + propranolol	Sinus bradycardia, hypotension	Probable	Grade 1, 3 (1)	50	Propranolol discontinuation, hydration, inotropic drugs
Bosentan + sildenafil^a^	Alanine aminotransferase increased	Probable	Grade 3 (1)	20	Bosentan discontinuation
Ciprofloxacin + propranolol	Sinus bradycardia, hypotension	Probable	Grade 1, 3 (1)	100	Propranolol discontinuation, hydration, inotropic drugs
Pentamidine + sulfamethoxazole/trimethoprim	Electrocardiogram QT corrected interval prolonged	Probable	Grade 3 (1)	50	Sulfamethoxazole/trimethoprim discontinuation
Desmopressin + dexamethasone	Hyponatremia	Probable	Grade 4 (1)	100	Desmopressin discontinuation, dexamethasone dosage reduction
Furosemide + propranolol	Sinus bradycardia, hypotension	Probable	Grade 1, 3 (1)	33.3	Propranolol discontinuation, hydration, inotropic drugs
Lansoprazole + warfarin	INR increased	Probable	Grade 2 (1)	100	Warfarin dosage adjustment after lansoprazole discontinuation
Dexamethasone + vecuronium	Decreased vecuronium effectiveness^b^	Possible	N/A (1)	25	Vecuronium dosage increase
Esomeprazole + levothyroxine^a^	Hypothyroidism	Possible	Grade 2 (1)	16.7	Levothyroxine dosage increase
Midazolam + voriconazole	Depressed level of consciousness	Possible	Grade 3 (1)	100	Midazolam dosage reduction
Furosemide + vecuronium^a^	Decreased vecuronium effectiveness^b^	Possible	N/A (2)	15.4	Vecuronium dosage increase
Phenobarbital + remifentanil^a^	Depressed level of consciousness	Possible	Grade 3 (1)	16.7	Remifentanil dosage reduction
Baclofen + remifentanil	Depressed level of consciousness	Possible	Grade 3 (1)	100	Remifentanil dosage reduction
Digoxin + norepinephrine	Atrial flutter, atrial fibrillation	Possible	Grade 3 (1)	100	Norepinephrine dosage reduction, cardioversion
Dopamine + digoxin	Atrial flutter, atrial fibrillation	Possible	Grade 3 (1)	33.3	Dopamine dosage reduction, cardioversion
Iron + lansoprazole	Anemia	Possible	Grade 2 (1)	20	Change lansoprazole to famotidine

^a^Listed in most frequently identified potential drug–drug interactions.

^b^Not available in Common Terminology Criteria for Adverse Events v5.0.

^c^Ratio of number of clinically relevant potential drug–drug interactions compared to the number of exposures for each drug pair.

### Factors associated with PDDIs and CR-PDDIs

The association between patient characteristics and the risk of PDDI exposure is presented in Table 5. In the univariate logistic regression analysis, sex, age (2–5 years), pediatric cardiology and thoracic surgery department, the average number of prescribed medications per person, and length of PICU stay were significantly associated with PDDIs. However, in the multivariate logistic regression analysis, only the number of prescribed medications and length of PICU stay showed a statistically significant association with PDDI exposure. Compared with patients administered an average of ≤5 medications per day, Patients receiving 6–7 (OR 5.6, 95% CI 1.6–19.1, *p =* 0.006) and >8 medications per day (OR 36.9, 95% CI 9.8–138.9, *p<*0.001) were more likely to be exposed to PDDIs than patients treated with an average of ≤5 medications per day. Moreover, compared with patients who stayed in the PICU for <1 week, those who stayed for 1–2 weeks had a five-fold higher likelihood of PDDI exposure (*p =* 0.035), and those who stayed for >2 weeks had an eight-fold higher likelihood of PDDI exposure (*p =* 0.049).

**Table 5:** Risk factors associated with potential drug–drug interactions and clinically relevant potential drug–drug interactions.

	All PDDIs^a^				Clinically relevant PDDIs^b^			
Variables	Univariate analysis		Multivariate analysis		Univariate analysis		Multivariate analysis	
	OR (95% CI)	*p*-value	OR (95% CI)	*p*-value	OR (95% CI)	*p*-value	OR (95% CI)	*p*-value
Sex								
Female	Reference		Reference		Reference			
Male	2.2 (1.1–4.5)	0.028	2.3 (0.8–6.4)	0.105	0.9 (0.3–2.7)	0.933		
Age (years)								
<2	Reference		Reference		Reference			
2–5	3.8 (1.1–13.6)	0.039	3.8 (0.7–19.9)	0.111	1.4 (0.3–5.6)	0.656		
6–11	2.9 (0.6–13.6)	0.186	3.2 (0.4–25.2)	0.268	1.8 (0.4–9.7)	0.475		
12–18	0.8 (0.3–2.1)	0.714	0.9 (0.2–4.2)	0.963	1.7 (0.4–7.1)	0.445		
Department								
General/Neurosurgery	Reference		Reference		Reference			
General pediatrics	2.1 (0.9–4.7)	0.091	0.6 (0.2–2.0)	0.357	3.2 (0.7–15.5)	0.155		
Cardiothoracic department^c^	4.9 (1.9–13.0)	0.001	1.3 (0.3–5.0)	0.708	1.9 (0.3–10.1)	0.471		
Complex chronic condition								
0–1	Reference				Reference^d^			
2–3	1.9 (0.9–4.1)	0.103						
≥4	1.8 (0.7–4.9)	0.243			5.5 (1.9–16.2)	0.002	4.4 (1.3–14.2)	0.015
Reason for PICU admission								
Post-operation/procedure	Reference				Reference			
Respiratory failure	0.8 (0.3–1.8)	0.549			1.9 (0.5–7.3)	0.334	1.0 (0.2–4.7)	0.928
Mental change	0.5 (0.1–2.5)	0.406			2.3 (0.2–22.7)	0.465	1.3 (0.1–15.5)	0.843
Shock	1.5 (0.3–7.8)	0.601			1.6 (0.2–14.4)	0.697	0.7 (0.1–8.5)	0.74
CRRT	1.9 (0.2–17.3)	0.559			7.0 (1.1–46.3)	0.043	1.8 (0.2–17.4)	0.626
Other causes	2.1 (0.4–10.2)	0.351			2.6 (0.5–14.2)	0.287	1.3 (0.2–10.8)	0.809
Predicted mortality rate^e^	1.0 (0.9–1.1)	0.214			1.0 (0.9–1.0)	0.512		
Prescribed medications^f^								
≤5	Reference		Reference		Reference^d^		Reference^d^	
6–7	7.7 (2.7–22.1)	<0.001	5.6 (1.6–19.1)	0.006				
≥8	52.8 (16.4–170.7)	<0.001	36.9 (9.8–138.9)	<0.001	14.8 (1.9–115.0)	0.01	12.3 (1.5–99.1)	0.019
Length of stay in PICU								
<1 week	Reference		Reference		Reference^d^		Reference^d^	
1–2 weeks	3.8 (1.7–10.7)	0.011	4.5 (1.1–17.9)	0.035				
≥2 weeks	6.3 (1.4–28.3)	0.017	7.7 (1.0–59.1)	0.049	6.2 (2.0–18.9)	0.001	3.4 (1.1–11.7)	0.048

CRRT, continuous renal replacement therapy; PDDI, potential drug–drug interaction; PICU, pediatric intensive care unit.

^a^Hosmer-Lemeshow goodness-of-fit test: *p =* 0.619, ^b^Hosmer-Lemeshow goodness-of-fit test: *p =* 0.692.

^c^Included departments of pediatric cardiology and thoracic surgery.

^d^Two consecutive top groups were merged owing to the small number of patients in each group.

^e^Estimated based on the Pediatric Risk of Mortality Score IV.

^f^Average number of prescribed medications by the PICU (maximum 30 days) per person.

The factors related to CR-PDDIs are presented in Table 5. In the univariate logistic regression analysis, the number of CCCs, reasons for PICU admission (for continuous renal replacement therapy), the average number of prescribed medications per patient, and length of PICU stay were significantly correlated with CR-PDDIs. However, the only independent variables that significantly affected the CR-PDDI occurrence in the final model based on the multivariate analysis were ≥4 CCCs (OR 4.4, 95% CI 1.3–14.2, *p =* 0.015), ≥8 prescribed medications (OR 12.3, 95% CI 1.5–99.1, *p =* 0.019), and a PICU stay of ≥2 weeks (OR 3.4, 95% CI 1.1–11.7, *p =* 0.048).

## Discussion

In this study, we characterized CR-PDDIs according to their severity, causality, and standard terminology by focusing on the clinical significance of identified PDDIs. Furthermore, we determined the predictors of CR-PDDIs and PDDIs. To date, there are only a few studies on the prevalence, common drug combinations, and risk factors of PDDIs in critically ill children [5,6,18,19]. However, studies on the negative effect or intensity of expected PDDIs are limited. To our knowledge, this study is the first to assess the clinical significance of PDDIs from the perspective of pediatric intensivists.

In the present study, although the prevalence of PDDIs was 72.3% in our patient population, CR-PDDIs were observed in only 10.1% of the patients. The prevalence of PDDIs was considerably higher in our study than in previous studies because we included only patients hospitalized for ≥3 days. Therefore, the prevalence of CR-PDDIs is expected to decrease further when all patients are included. This result is consistent with studies in adult ICUs, where only a few CR-PDDIs were observed among numerous PDDIs [4,20]. Despite the low prevalence in the present study, more than 50% of the CR-PDDIs exhibited a severity of grade ≥3 and required immediate management. Moreover, CR-PDDIs occurred between drugs used less frequently than between drugs involved in common PDDIs. According to a report by Dai et al., exposure to the most common PDDIs may not pose the greatest risk for patients because clinicians may be familiar with and prepared to manage the DDIs [5]. Furthermore, it was found that most of the potential ADRs could be detected early or prevented by routine intensive care in PICU. For example, most of the common PDDIs can be actively monitored by frequently checking vital signs, performing blood tests that include drug level analysis, controlling the hourly urine output, and regularly assessing the sedation depth, which will ensure the detection of actual ADRs [21]. As seen in the drug combinations involved in PDDIs in our study, most of the drugs were anti-infective, cardiovascular, and central nervous system agents, which are high-risk medications known to develop DDIs according to previous studies [22–25]. Nevertheless, intensivists cannot avoid prescribing these high-risk medications as they are essential for managing and treating diseases requiring intensive care. Therefore, in this special environment, the basic management in ICUs can play an important role in monitoring PDDIs. Furthermore, it is presumed that the level of care in PICUs can ultimately affect CR-PDDIs.

Remarkably, the prevalence of PDDIs and CR-PDDIs did not differ according to the department to which the patients belonged. However, drug combinations of frequently identified PDDIs varied with the department. Similar to our results, differences in the pattern of PDDIs have been reported, especially in adult ICU patients, including cardiothoracic patients [24,25]. The common drug combinations associated with PDDIs also differ between PICUs and adult ICUs. For example, despite the heterogeneity of patients included in the studies, aspirin, insulin, and clopidogrel are the most common drugs implicated in DDIs in adult ICUs [4], whereas these drugs are not commonly used in pediatric patients. Furthermore, even among pediatric patients, frequent drug combinations related to PDDIs differ between hospitals [5,6,18,19]. This is presumed to be due to differences in the composition of critically ill patients, drug preferences of intensivists, and drug permits in each country. In South Korea, remifentanil is permitted for off-label use in PICU patients requiring mechanical ventilation. Consequently, remifentanil with midazolam is a common drug pair causing PDDIs. Therefore, we suggest that high-risk drugs requiring monitoring of PDDIs should be selected depending on the patients admitted to the PICU.

Polypharmacy and prolonged hospitalization in ICU are well-known risk factors of PDDIs in ICUs, [5,6,24,25]. The results of this study are consistent with those of previous studies; however, we discovered that these risk factors eventually increase the likelihood of “actual” DDIs and not just “potential” DDIs. These two risk factors are not considered independent. Because the daily exposure to drugs increases with increase in the length of PICU stay [5], prolonged hospitalization is estimated to indirectly increase the risk of DDIs through polypharmacy. Consequently, the number of medications is the most important predictive factor of whether PDDIs will lead to actual ADRs. In addition, comorbidities were also revealed to increase the risk of PDDIs not only in adult ICUs but also in PICUs [5,22]. However, in the present study, the CCCs were significantly associated only with a higher prevalence of CR-PDDIs and not with PDDIs. Here, because we had a smaller sample size than the previous studies, it was not possible to identify the relationship between CCCs and PDDIs. Despite this limitation, we demonstrated that patients with multiple chronic conditions were at a higher risk of CR-PDDIs, and thus, special attention is required.

Finally, to limit the actual manifestation of PDDIs, clinicians should be aware of the probability of DDI occurrence, based on the characteristics and risk factors of CR-PDDIs. In our hospital, clinical pharmacists were recently introduced in the PICU, and it has been demonstrated that they can contribute to efficient and safe pharmacotherapy, including DDI monitoring, in the ICU setting [10,20,26]. However, due to the limited number of clinical pharmacists, not all drugs administered in PICUs can be manually monitored. To overcome this problem, a clinical decision support system integrated with the electronic health records was developed. This system is expected to prevent adverse events including DDIs through automatic monitoring and alerts in hospital. Nevertheless, concerns about frequent alert fatigue with clinically insignificant DDIs have been raised, since the monitoring system are based on large-scale common data [27]. Consequently, electrical and automatic monitoring systems are not enough to prevent significant DDIs, especially in ICUs where drugs with a high probability of DDIs are frequently prescribed and DDIs actually occur between infrequently used drugs. Therefore, without relying only on monitoring systems, clinicians, ‘themselves’, should pay attention to CR-PDDIs whether they prescribe commonly or rarely used drugs. Furthermore, we recommend preemptive investigation of patient characteristics commonly encountered in their respective PICUs, drugs used frequently in each hospital, and the status of CR-PDDIs as well as PDDIs. Subsequently, an appropriate monitoring plan for comprehensive and effective surveillance of ADRs caused by PDDIs could be developed according to the results and modified depending on each PICU environment.

This study has some limitations. First, although the chosen research institute is one of the major tertiary hospitals in South Korea with a large-scale PICU with 24 beds, the study was conducted in a single institution and restricted to a short period of 6 months, limiting the representation and generalization of the findings. Thus, a multicenter study with a longer study period is required to obtain more concrete information. Second, because we conducted a retrospective study, there might be an underreporting of some ADRs. Nevertheless, since clinical pharmacists assigned to our PICU had to screen PDDIs, the data about drug use and DDIs could be considered to be collected prospectively. Third, because all the drugs administered in the PICU were included in this study regardless of where the drug was first initiated, the timing of CR-PDDI detected could not considered. To provide efficient guidance for monitoring PDDI, it is necessary to conduct further research regarding when CR-PDDI occurs following PDDI identification. Fourth, although the high accuracy of the Micromedex Drug-Reax^®^ system has been proven, the information about the pediatric population is limited because there is a lack of studies in pediatric settings. Additionally, some drug types could not be searched in this system due to a lack of information; thus, the associated PDDIs have to be confirmed later.

## Conclusion

In conclusion, a large proportion of patients in PICUs are exposed to PDDIs, but most of those do not appear as actual DDIs under routine intensive care. However, once CR-PDDIs occur, they are of high severity. Hence, there is an urgent need to develop a monitoring system for preventing CR-PDDIs via PDDI identification, especially based on the characteristics of the PICU.

## Supporting information

S1 Appendix Unsearched drugs in the Micromedex Drug-Reax^®^. (DOCX)

S2 Appendix Data underlying the results described in this study. (SAV)
