# Supplementary material for: Clinical significance of potential drug–drug interactions in a pediatric intensive care unit: A single-center retrospective study
Source: PLoS One. 2021 Feb 8;16(2):e0246754. doi: 10.1371/journal.pone.0246754 (PMC7870058; doi:10.1371/journal.pone.0246754)
Supplement: S1 Appendix — (DOCX) [file pone.0246754.s001.docx]

**S1 Table. Unsearched drugs in the Micromedex Drug-Reax^®^**

| **Number** | **Brand name** | **Generic name** |
| --- | --- | --- |
| **1** | UDCA | Ursodeoxycholic acid |
| **2** | Ramnos | *Lactobacillus casei* |
| **3** | Smecta | Dioctahedral smectite |
| **4** | Pheburane granule | Sodium phenylbutyrate |
| **5** | Flumarin | Flomoxef |
| **6** | Erdos | Erdosteine |
| **7** | Primalan | Mequitazine |
| **8** | Synatura | Ivy leaf, *Coptis* Rhizome extract |
| **9** | Bioflor | *Saccharomyces boulardii* |
| **10** | Nasea | Ramosetron |
| **11** | Neutrogin | Lenograstim |
| **12** | Onon cap | Pranlukast |
| **13** | Itomed | Itopride |
| **14** | Motilitone | *Corydalis* tuber, *Pharbitis* seed |
